# Removal of Ochratoxin A from Red Wine Using Alginate-PVA-*L. plantarum* (APLP) Complexes: A Preliminary Study

**DOI:** 10.3390/toxins14040230

**Published:** 2022-03-22

**Authors:** Ricardo Ignacio Castro, V. Felipe Laurie, Carlos Padilla, Verónica Carrasco-Sánchez

**Affiliations:** 1 Multidisciplinary Agroindustry Research Laboratory, Instituto de Ciencias Químicas y Aplicadas, Universidad Autónoma de Chile, Talca 3467987, Chile; ricardo.castro@uautonoma.cl; 2 Facultad de Ciencias Agrarias, Universidad de Talca, Talca 3460000, Chile; flaurie@utalca.cl; 3 Departamento de Microbiología, Facultad de Ciencias de la Salud, Universidad de Talca, Talca 3460000, Chile; cpadilla@utalca.cl

**Keywords:** mycotoxins, ochratoxin A, lactic acid bacteria, polyvinyl alcohol, alginate

## Abstract

The presence of ochratoxin A (OTA) in wines is a problem mainly due to the health damage it can cause to frequent drinkers. A method for removing these toxic substances from wine is the use of lactic acid bacteria with mycotoxin-adsorption capacities; however, their use is limited since a matrix in which they can be immobilized, to remove them after use, is needed. In this study, *L. plantarum* (LP) was encapsulated in a polymeric matrix composed of polyvinyl alcohol (PVA) and alginate, forming alginate–PVA–LP (APLP) complexes. Then, these complexes were characterized, and assays of OTA and phenol removal from wines were performed. As a result, it was observed that the APLP complexes at a concentration of 0.5 g mL^−1^ removed over 50% of the OTA without substantially affecting the concentration of total phenols. In addition, it was determined that the presence of *L. plantarum* directly affected the ability to adsorb OTA from wines and did not decrease the total phenols. In conclusion, an alginate–PVA matrix allows immobilizing LP, and the complexes formed are an alternative for removing ochratoxin from contaminated wines.

## 1. Introduction

Ochratoxin A (OTA) is a mycotoxin produced mainly by *Aspergillus carbonarius*, *A. ochraceus*, *A. niger*, *Penicillium verrucosum*, and *P. nordicum* [1]. Structurally, it consists of a p-chlorophenolic group linked to a dihydroisocoumarin fragment linked by an amide bond to an L-phenylalanine [2]. Additionally, it has been found as a contaminant in cereals, beer, coffee beans, cacao, spices, dried wine fruit, grape juice, and wine, and human blood and animal tissues [3].

The presence of OTA in wines was first reported in Switzerland in 1996 [4]. Since that first report, this mycotoxin has been described in wines in several countries worldwide [5,6,7].

The contamination of wines with OTA should raise a public health alert worldwide for frequent drinkers since these toxins can cause acute to chronic poisoning [8,9,10], and wine is considered the second most important dietary source of OTA after cereals [11]. Thus, the regulation 1881/2006 of the European Commission (EC) established a concentration of 2 µg kg^−1^ as the maximum tolerable level in wines destined for human consumption [12].

Many scientists have focused on studying different strategies (physical, chemical, and biological) to remove mycotoxins from red wines [13,14,15,16]. Although many chemical strategies have been promising, their use is limited by possible side effects and is not allowed within the EC for commodities destined for human consumption [3]. Therefore, biological agents are an alternative, mainly due to the ability of lactic acid bacteria (LAB) to adsorb mycotoxins in aqueous matrices [17,18,19,20]. The most versatile LAB seem to be *Lactobacillus* species, which efficiently remove OTA [19,21].

Using free microbial cells can present tremendous stresses such as those regarding survival, proliferation, mechanical disturbances, low adaptation, and competition from indigenous microorganisms in natural environments [22]. Immobilization strategies emerge as an alternative solution since immobilized bacteria can be shielded from the stress of high pollutant concentrations, predators, and competition with indigenous microorganisms [23].

This study proposes a new system (APLP complex) composed of lactic acid bacteria with known mycotoxin-adsorption activity encapsulated in a matrix composed of alginate and polyvinyl alcohol (PVA) polymers as a strategy for mycotoxin removal from red wines.

## 2. Results and Discussion

### 2.1. Development and Characterization of Alginate–PVA–L. plantarum Complexes 

#### 2.1.1. Development of Alginate–PVA–*L. plantarum* Complexes

In the present study, a strain of *Lactobacillus plantarum* (LP) with known OTA-adsorbing capacity was encapsulated in alginate–PVA (AP) polymers, forming alginate–PVA–*L. plantarum* (APLP) complexes, to develop a handy and easily removable tool for adsorbing the mycotoxin from red wines. 

**Figure 1:**
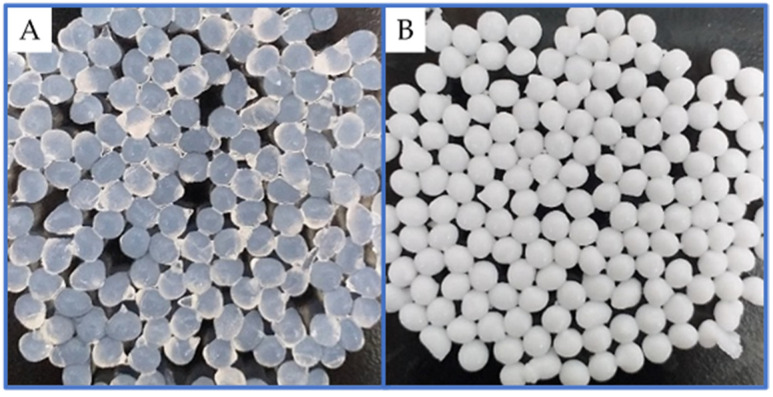
Macroscopic structure of (**A**) alginate, and (**B**) APLP complexes.

Alginate was used because it is a natural polymer (composed of D-mannuronic acid and D-glucuronic acid), non-toxic, biocompatible, and biodegradable, and is exploited in the food industry. Even in the wine industry, it is studied for encapsulating living yeast cells to carry out controlled fermentation [24]. Thus, *L. plantarum* was encapsulated in a polymeric matrix composed of PVA linked to boric acid (BA) and glutaraldehyde (GA); and alginate (Alg)’s crosslinking with calcium ions and the microspheres presented a sub-spherical shape measuring 1.5–2 mm in diameter (Figure 1). The complexes developed and their compositions are detailed in Table 1.

**Figure 2:**
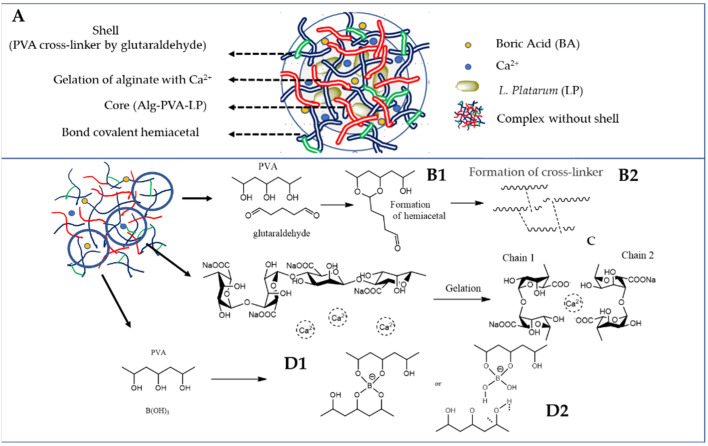
(**A**) APLP complex; (**B1**) formation of hemiacetal; (**B2**) PVA crosslinked with glutaraldehyde; (**C**) gelation mechanism known as an egg-box model; (**D1**) formation of PVA–BA complex; and (**D2**) hydrogen bonding between the diol complex and PVA.

The proposed mechanism for developing APLP complexes consisted of the following steps: first, the BA acts as a crosslinker for PVA, forming dioxaborinane rings [25], followed by hemiacetal formation between GA and the OH groups on PVA [26]. Second, the encapsulation of *L. plantarum* in calcium alginate networks, induced by the gelation resulting from specific and strong ionic interactions between Ca^2+^ and G blocks of alginate, results in the “egg-box” structure [27,28] (Figure 2).

Even though PVA can form spheres when crosslinked with boric acid, these tend to agglomerate, mainly due to the relatively slow crosslinking. For this reason, a mixed solution of PVA and sodium was used. In this way, it was thought that the PVA could improve the durability and resistance of the pearls, while the calcium alginate could improve the surface properties of the pearls, reducing the tendency to agglomeration [29].

On the other hand, it was also shown that the polymeric matrix prevented the release of bacteria when the complexes were placed in contact with a model solution of wine at pH 3.5. The prior allows us to suspect that these complexes have good stability and could possibly be used in wine, given that no cells that could compromise wine composition were being released. In any case, the prior requires further studies to confirm this observation under different environmental conditions.

#### 2.1.2. Characterization of Alginate–PVA–*L. plantarum* Complexes

**Figure 3:**
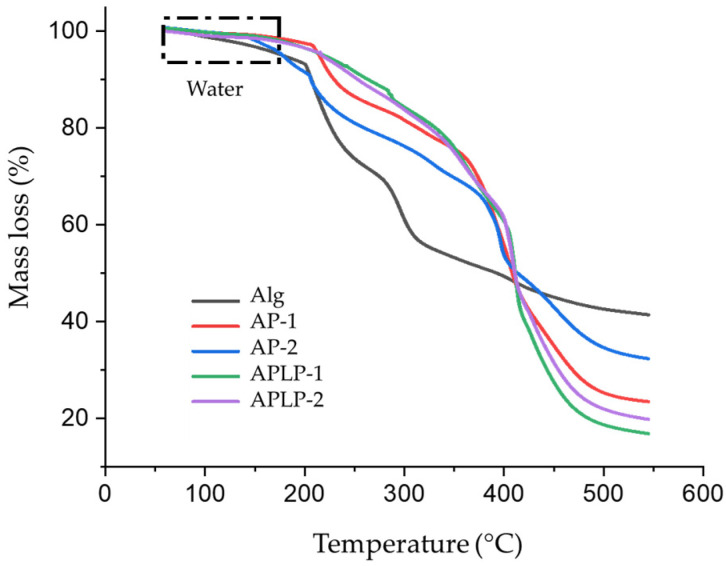
Thermal gravimetry curves of Alginate (Alg), AP−1, AP-2, APLP−1, and APLP-2 complexes.

The characterization of the alginate, AP, and APLP complexes by thermogravimetry analysis (TGA) (Figure 3) showed that all the samples had the capacity for water absorption; this result is observed in the regions of the TGA curves between 50 and 180 °C, which is indicative of the loss of moisture and suggests either physically weakly or chemically strongly bound water [30,31]. 

However, the formation of a crosslinker (boric acid) between the chain of PVA and the gelation of alginate with calcium ions decreases the water absorption and increases the thermal stability of the complex due to the interaction of PVA chains and the possible ionic interactions between the hydroxyl groups of both chains (double-network hydrogels). Additionally, the increase in the thermal stability of the complex may also be due to the change in molecule between B(OH)_3_ and water [32].

Finally, the APLP−1 and APLP-2 curves indicate increased stability and decreased water absorption, possibly due to the interaction of *L. plantarum* with the alginate and PVA chains, which suggests the formation of the complex.

### 2.2. OTA Removal from Red Wines for APLP Complexes

#### 2.2.1. Study of Effects of Complex Concentration on OTA and Phenol Removal

**Figure 4:**
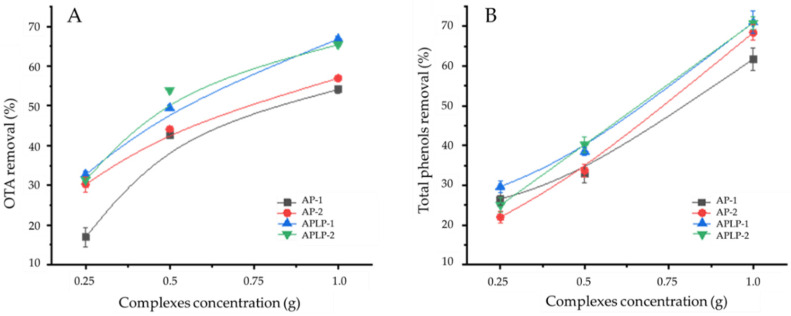
Influence of complex concentration on removal of (**A**) OTA and (**B**) phenols.

The kinetic curves showed that APLP−1 and APLP-2 at a concentration of 1 g mL^−1^ removed over 60% of the OTA. However, it also removed over 60% of the total phenols. Therefore, to obtain a removal result of over 50% for OTA without substantially affecting the concentration of total phenols, we concluded that the best concentration was 0.5 g mL^−1^ (Figure 4).

#### 2.2.2. Optimization of OTA and Phenol Removal by APLP Complexes

An optimization assay was performed to determine the best microcapsule formation with affinity for OTA using a statistical model of 2^3^ through a factorial analysis designed in the Statgraphics Centurium XVI program. The values obtained for OTA removal while phenols were not removed are shown in Table 2.

Regarding the OTA and phenol removal by the AP and APLP complexes, the analysis of the variance of the mathematical models obtained from the results yielded the coefficients of the response function for the dependent variables, which were determined using Statgraphics Centurium XVI. Table 3 shows the statistically significant factors, and the correlation of the models for the estimated responses.

For OTA, different response functions are described in Equation (1) for the response surface plot, which shows the complete regression model (*R*^2^ = 93.38; standard error = 1.349) obtained for the removal. Based on the regression coefficient, this model explained 93% of the responses observed in the assays.

(1) $$OTA\;removal\;\left(\%\right)=48.8701+4.744449A+5.17880B+0.317578C\;-\;1.80774A^{2}+0.0086863AB\;+1.54014AC-3.98695B^{2}+0.00683227\;B$$

The analysis of the capture of phenols, expressed as non-captured phenols, was performed based on Equation (2) from the response surface plot, producing the complete regression model (*R*^2^ = 91.51; standard error = 1.239). Based on the regression coefficient, this model explained 92% of the responses observed in the assays.

(2) $$Phenols\;not\;catched\;\left(\%\right)=60.2384\;-0.996408\;-2.0083B\;-\;2.8967C\;+\;1.23333A^{2}+0.52137AB\;-0.31111AC+3.13105B^{2}+0.385037\;BC$$

**Figure 5:**
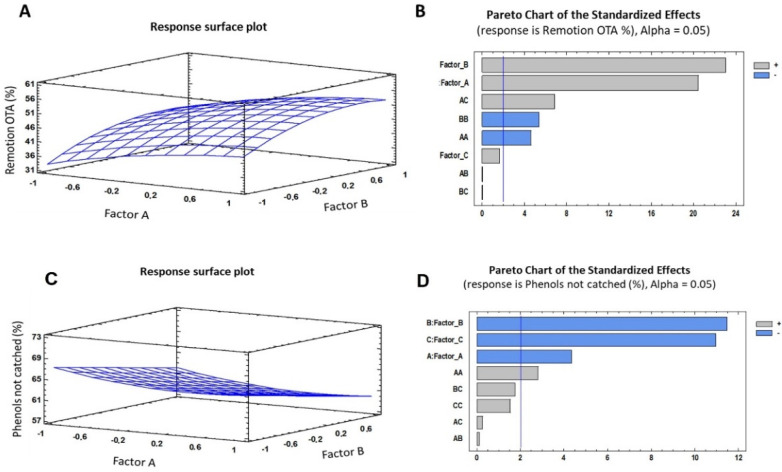
(**A**,**C**) Graphics for the estimated response surface for OTA removal (%) and phenols unremoved respectively; (**B**,**D**) Standardized Pareto chart for OTA removal (%), and phenols unremoved. Factor A: LP concentration, Factor B: Exposition time (min), and Factor C: Molecular weight of PVA. Blue line represents critical *t*-value, 95% confidence.

The optimization for the capture of OTA and the conservation of the phenols in solution (Table 2) revealed the combination of the levels of the factors that maximized the responses for the parameters studied. For the capture and removal of OTA, it was found that the most important factors were time and concentration (Figure 5). 

For *L. plantarum*, it is shown that its presence directly affects absorption, increasing the ability to remove OTA from wine by approximately 8%; these data agree with the studies reported by de Prete et al. (2007) [33], who studied different strains of *Lactobacillus*, which were capable of removing 8 to 28% of the OTA from wine depending on the strain. The theoretical analysis showed that an exposure time of 52 min was necessary to maximize the removal. These data are complemented by the thermograms (Figure 3), in which it was observed that the presence of LAB increased the system’s stability.

The mechanism by which lactic acid bacteria are capable of adsorbing OTA is believed to be the adsorption of these toxins to the surface structures of the cell wall, where peptidoglycan and exopolysaccharides would play an important role [34]. These interactions could be promoted by the hydrophobic properties of the cell wall and electron donor–acceptor and Lewis’s acid–base interactions [35].

Furthermore, the inclusion of LP in the complex facilitates OTA removal without significantly affecting phenol removal. Thus, for example, it was observed that the removal of phenols by the complex without LP at one hour of contact using AP−1 (PVA Mw ∼47,000) and AP-2 (PVA Mw ∼61,000) was 64.17 and 61.87, respectively; however, when BAL (APLP−1 and PLP-2) were added to the complexes, the removal of phenols only increased by 0.5% and 2.53%, respectively. Additionally, more thermally stable complexes were generated. 

Finally, there is no doubt that the best strategy is the prevention of contamination of raw materials with fungi. However, to date, total management in the field is not possible, and removal strategies are an alternative to mitigate the final problem, as long as they go hand in hand with adequate detection strategies [36].

## 3. Conclusions

The APLP complexes were efficient in removing OTA from wines without substantially affecting their phenolic quality, and theoretically, a time of only 52 min was necessary to achieve the objective of removing over 50% of the OTA. Additionally, the presence of *L. plantarum* in the complexes increased the OTA-removal capacity without affecting the phenolic composition of the wine. 

It should be considered that this was a screening study, and future studies should consider evaluating the removal of phenols individually to determine how this process affects each of them. Finally, the possibility of immobilizing lactic acid bacteria in polymeric matrices that are approved for use in the food industry is an important aspect to consider in advancing these strategies for removing mycotoxins from wines and obtaining safer beverages that do not cause damage to health.

## 4. Materials and Methods

### 4.1. Chemicals and Medium

Sodium alginate, mowiol™ 6-98 (PVA 6-98; polyvinylalcohol, Mw ∼47,000, 98% hydrolyzed), mowiol™ 10-98 (PVA 10-98; polyvinylalcohol, Mw ∼61,000, 98% hydrolyzed), calcium chloride anhydrous (CaCl_2_, ≥96.0%), gallic acid (≥95.0%), and boric acid (B(OH)_3_, ≥99.5%) were obtained from Sigma-Aldrich (St. Louis, MO, USA). 

Glutardialdehyde (GA, 50% solution in water), absolute ethanol, L(+)-tartaric acid (≥99.5%), sodium hydroxide (NaOH, ≥99%), chloride acid (HCl), De Man Rogosa Sharpe (MRS) medium, solvents for HPLC analysis of chromatographic grade (ultra-pure water, H_2_O; acetonitrile, ACN; acetic acid, CH_3_COOH), Folin-Ciocalteu’s phenol reagent (≥99.5%; 2 N), and PTFE membrane filters (0.45 µm) were purchased from Merck KGaA (Darmstadt, Germany). Deionized water from a Millipore Milli-Q-P Plus system was used for preparing the aqueous solutions and for HPLC analysis. 

QuEChERS extraction kits (salts—1 g of sodium citrate, 0.5 g of disodium citrate sesquihydrate, 4 g of magnesium sulfate (MgSO_4_), and 1 g of sodium chloride—and dispersive solid-phase extraction tubes, 900 mg of MgSO_4_, and 150 mg of primary secondary amine (PSA) sorbent) were purchased from Agilent Technologies (Santa Clara, CA, USA).

Ochratoxin A (from *Petromyces alberiensis*, ≥98%), was obtained from Sigma-Aldrich (St. Louis, MO, USA), and the stock solution (1 mg mL^−1^ in absolute ethanol) was stored at −20 °C. 

### 4.2. Bacterial Strain and Culture Condition

The *Lactobacillus plantarum* 299v (LP) strain was obtained commercially from BION, Merck. Prior to use, the LP was stored frozen in MRS broth with 15% (*v*/*v*) glycerol. For testing, the LP were activated in MRS broth under incubation at 37 °C for 24 h in a 5% CO_2_ incubator, subculturing in MRS agar. Then, to obtain wet cells, *L. plantarum* were incubated in 500 mL Erlenmeyer flasks containing 300 mL of MRS broth at 37 °C in anaerobic conditions. After incubation for 24 h, the wet cells were collected by centrifugation at 10,000 rpm at 4 °C for 10 min [37], and the cell pellet was collected and immediately washed thrice with 0.9% sterilized sodium chloride solution [22].

### 4.3. Immobilization of L. plantarum in Alginate-PVA and Bacterial Delivery Assay 

Alginate–PVA–*L. plantarum* (APLP) complexes were prepared according to the modified methodologies used by Long et al. (2004) [29] and Many et al. (2019) [38]. First, a 2% alginate (Alg) solution with 5% PVA (Mw ∼47,000 or ∼61,000) was prepared by dissolving alginate in hot distilled water and autoclaving it at 121 °C for 15 min. Then, the cell suspension containing wet cells (1 or 2 g) was added to 100 mL of Alg–PVA (AP) solution and stirred for 10 min to produce a homogeneous slurry. The mixture obtained was taken into a sterile syringe and extruded dropwise into a sterile solution composed of 10% boric acid and 2% CaCl_2_ (crosslinking agent) to form complexes. In order to complete the gelation, these complexes were kept in a 5% BA solution for 24 h at 4 °C. Finally, the complexes were removed and washed with distilled water. The formulation of the synthesized complexes is detailed in Table 1. 

In order to determine the bacteria released from the polymeric matrix, 1 g of APLP−1 and APLP-2 complexes with 1 mL of a model wine solution (consisting of 12.5% ethanol solution (*v*/*v*), adjusted to pH 3.5 using tartaric acid (5.0 g L^−1^) and a 1 mM solution of sodium hydroxide) were put in contact during 60 min. After that contact time, serial dilutions were made and seeded on count agar plates to count colonies.

### 4.4. Complex Characterization for Thermal Analysis by Thermogravimetry (TGA)

The alginate, AP−1, AP-2, APLP−1, and APLP-2 complexes were characterized using a thermogravimetric analyzer: STD 650 TA-instruments. The samples were heated at a constant heating rate of 10 °C min^−1^. Heating from room temperature to 900 °C was realized in air, a reactive gas, with a mass flow of 50 mL min^−1^. Additionally, N_2_ (50 mL min^−1^) was used as a protection gas in the electronic balance. A 5 mg amount of the mixture was placed into a Pt crucible for each analysis.

### 4.5. OTA Removal from Red Wines by AP and APLP Complexes 

The first OTA-removal assay was carried out to determine the concentration of complexes that would allow at least 50% of the OTA to be removed without the total phenol concentration being substantially impaired. For this, 0.25, 0.5, and 1.0 g of AP−1, AP-2, APLP−1, and APLP-2 were weighed into 12 mL glass tubes; then, 1.0 mL of red wine (Cabernet Sauvignon 2019, OTA free), spiked with 5000 ng L^−1^ of OTA, was added. The samples were mixed and agitated for 60 min at room temperature (20 °C), using a rock motion agitator, operating at 100 rpm. Then, the complexes and wines were separated, and the concentration of free OTA in the red wine was analyzed (see Section 4.6).

For the second OTA-removal assay, a factorial design (3^3^) with two variables and three levels was established. Runs were performed randomly to optimize the time, bacterial concentration, and molecular weight for the PVA. The nine designed experiments were carried out in triplicate; the experimental factors (uncoded units) were transformed into coded units and coded as −1, 0, and +1. The response was expressed as the percentage of OTA removal. The data were analyzed by the analysis of variance (ANOVA), with a significance level of 95% (*p* ≤ 0.05).

For this, 0.5 g of each complex (Table 1) was weighed into 12 mL glass tubes, followed by adding 1.0 mL of red wine (Cabernet Sauvignon 2019, OTA free), spiked with 5000 ng L^−1^ of OTA. The samples were mixed and agitated for different times (15, 30, and 60 min) at room temperature (20 °C, 100 rpm). Then, the complexes and wines were separated, and the concentration of free OTA in the red wine was analyzed (see Section 4.6). The controls used were control 1 (1% LP), control 2 (2% LP), control 3 (Alg complex), control 4 (Alg–1% LP complex), and control 5 (Alg–2% LP complex). 

In addition to the OTA analyses, the total phenolic concentration was determined using the Folin–Ciocalteu method [39], using a Spectroquant Pharo 300 UV–Visible spectrophotometer. The total phenolic concentration was estimated based on a standard curve of gallic acid (0–500 mg L^−1^).

### 4.6. Analysis of OTA

#### 4.6.1. OTA Extraction and Purification from Red Wine

For extraction and purification of the OTA from the wine, we used the methodology used by Carrasco-Sánchez et al. (2018) [40]. Briefly, 1 mL of red wine samples were extracted and partitioned with 2 mL of acetonitrile/acetic acid (99/1) and 0.5 g of QuEChERS extraction salts. The mixture was stirred (10 s) and centrifuged at 1500× *g* for 5 min. Then, the supernatant was extracted and cleaned using 900 mg of MgSO_4_ and 150 mg of PSA sorbent. The mixture was then stirred and centrifuged for 2 min at 1500× *g*, and the supernatant was filtered through PTFE membrane filters (0.45 µm), prior to OTA analysis by high-performance liquid chromatography with a fluorescence detector (HPLC-FLD).

#### 4.6.2. Analysis of OTA by HPLC-FLD

The OTA concentrations in the extracts obtained from the red wine samples were analyzed using an HPLC-FLD system (Agilent Technologies 1260 Infinity) equipped with a quaternary pump and autosampler. The separation was performed using a reverse-phase LiChrocart^®^ 250-4 RP−18 (250 mm × 4 mm ID × 5 μm) column (Merck), under the following conditions: a mobile phase consisting of H_2_O:ACN:CH_3_COOH (49.5:49.5:1, *v*/*v*), operated in isocratic mode, at a flow rate of 0.9 mL min^−1^. The injection volume was 40 μL, and the analyte detection was performed at Ex: 334 nm and Em: 460 nm [40].

## Figures and Tables

**Table 1:** Formulation of AP and APLP complexes.

Complexes	PVA	LP Concentration in Wet Weight
AP−1	5% Mowiol 6–98	-
APLP−1	5% Mowiol 6–98	1%
APLP−1b	5% Mowiol 6–98	2%
AP-2	5% Mowiol 10–98	-
APLP-2	5% Mowiol 10–98	1%
APLP-2b	5% Mowiol 10–98	2%

Note: AP: Alginate-PVA; APLP: Alginate-PVA-*L. plantarum.*

**Table 2:** Matrix of the experimental design for the variables; coded and uncoded values of the factors.

	Uncodified			Codified			Results	
Complexes	PVA (Mw)	LAB (%)	Time (min)	Mw PVA	% LAB	Contact Time	OTA Removal (%)	Unremoved Phenols (%)
AP−1	47,000	0	15	−1	−1	−1	32.22 ± 0.71	72.23 ± 0.61
APLP−1	47,000	1	15	−1	0	−1	38.99 ± 0.12	67.50 ± 1.65
APLP−1b	47,000	2	15	−1	1	−1	40.40 ± 0.79	68.13 ± 1.20
AP-2	61,000	0	15	1	−1	−1	33.94 ± 0.43	64.53 ± 0.49
APLP-2	61,000	1	15	1	0	−1	40.52 ± 0.53	62.40 ± 1.41
APLP-2b	61,000	2	15	1	1	−1	44.92 ± 0.31	62.41 ± 0.78
AP−1	47,000	0	30	−1	−1	−0.66	39.46 ± 0.28	67.73 ± 0.97
APLP−1	47,000	1	30	−1	0	−0.66	45.45 ± 0.44	67.23 ± 1.26
APLP−1b	47,000	2	30	−1	1	−0.66	45.25 ± 0.26	66.43 ± 2.29
AP-2	61,000	0	30	1	−1	−0.66	34.69 ± 0.29	62.90 ± 0.70
APLP-2	61,000	1	30	1	0	−0.66	42.62 ± 0.40	59.17 ± 0.60
APLP-2b	61,000	2	30	1	1	−0.66	47.59 ± 0.36	59.03 ± 0.57
AP−1	47,000	0	60	−1	−1	1	45.64 ± 0.11	64.17 ± 0.45
APLP−1	47,000	1	60	−1	0	1	48.48 ± 0.38	65.03 ± 0.64
APLP−1b	47,000	2	60	−1	1	1	50.87 ± 0.65	64.77 ± 0.51
AP-2	61,000	0	60	1	−1	1	41.71 ± 0.82	61.83 ± 1.12
APLP-2	61,000	1	60	1	0	1	50.91 ± 0.68	58.00 ± 0.46
APLP-2b	61,000	2	60	1	1	1	55.53 ± 0.79	59.30 ± 0.82

**Table 3:** Analysis of variance (ANOVA) from mathematical model for OTA removal and Phenols unremoved in wines.

	Source	Sum of Squares	Degree’s Liberty	Mean Square	Razon-F	Valor-P
OTA removal	A: Factor A	762.057	1	762.057	418.34	0.0000
	B: Factor B	965.55	1	965.55	530.05	0.0000
	C: Factor C	5.12154	1	5.12154	2.81	0.1008
	AA	39.215	1	39.215	21.53	0.0000
	AB	0.00207382	1	0.00207382	0.00	0.9732
	AC	85.3931	1	85.3931	46.88	0.0000
	BB	53.0585	1	53.0585	29.13	0.0000
	BC	0.00192448	1	0.00192448	0.00	0.9742
	Blocks	0.0981739	2	0.0490869	0.03	0.9734
	Total error	78.3301	43	1.82163		
Unremoved phenols	A: Factor A	33.611	1	33.611	21.86	0.0000
	B: Factor B	145.203	1	145.203	94.45	0.0000
	C: Factor C	426.116	1	426.116	277.17	0.0000
	AA	18.2533	1	18.2533	11.87	0.0013
	AB	7.47136	1	7.47136	4.86	0.0329
	AC	3.48444	1	3.48444	2.27	0.1395
	BB	32.7231	1	32,7231	21.28	0.0000
	BC	6.11208	1	6.11208	3.98	0.0525
	Blocks	4.77778	2	2.38889	1.55	0.2231
	Total error	66.108	43	1.5374		

## Data Availability

Not applicable.
